# Myoelectric-Based Estimation of Vertical Ground Reaction Force During Unconstrained Walking by a Stacked One-Dimensional Convolutional Long Short-Term Memory Model

**DOI:** 10.3390/s24237768

**Published:** 2024-12-04

**Authors:** Alessandro Mengarelli, Andrea Tigrini, Mara Scattolini, Rami Mobarak, Laura Burattini, Sandro Fioretti, Federica Verdini

**Affiliations:** Department of Information Engineering, Università Politecnica delle Marche, 60131 Ancona, Italy; a.tigrini@staff.univpm.it (A.T.); m.scattolini@pm.univpm.it (M.S.); r.mobarak@pm.univpm.it (R.M.); l.burattini@staff.univpm.it (L.B.); s.fioretti@staff.univpm.it (S.F.); f.verdini@staff.univpm.it (F.V.)

**Keywords:** ground reaction force, walking, regression, LSTM, EMG, prosthetic control

## Abstract

The volitional control of powered assistive devices is commonly performed by mapping the electromyographic (EMG) activity of the lower limb to joints’ angular kinematics, which are then used as the input for regulation. However, during walking, the ground reaction force (GRF) plays a central role in the modulation of the gait, providing dynamic stability and propulsion during the stance phase. Including this information within the control loop of prosthetic devices can improve the quality of the final output, providing more physiological walking dynamics that enhances the usability and patient comfort. In this work, we explored the feasibility of the estimation of the ground reaction force vertical component (VGRF) by using only the EMG activities of the thigh and shank muscles. We compared two deep learning models in three experiments that involved different muscular configurations. Overall, the outcomes show that the EMG signals could be leveraged to obtain a reliable estimation of the VGRF during walking, and the shank muscles alone represent a viable solution if a reduced recording setup is needed. On the other hand, the thigh muscles failed in providing performance enhancements, either when used alone or together with the shank muscles. The results outline the feasibility of including GRF information within an EMG-driven control scheme for prosthetic and assistive devices.

## 1. Introduction

In the last few decades, the usage of powered assistive and prosthetic devices for lower limb assistance has been promoted by technological developments in terms of both hardware and software that foster the actual exploitation of this kind of technology for impaired individuals [1]. In this field, a key role is played by the control schemes adopted for allowing the user to interact with the device [2], with the volitional control representing one of the favored ways to achieve the user’s direct regulation, allowing the powered prosthesis to tune its mechanical response to satisfy the intent of motion of the subject [3].

In order to establish a link between the user movement intention and the actual behavior of the device, it is necessary to build up a human–machine interface by choosing a suitable source of information at a low level, with the potential to be decoded by higher-level control structures to provide the final device modulation [1]. In this view, the muscular electrical activity, acquired by electromyography (EMG), serves as one of the preferred biosignals for enabling an active interaction between the user and the device [2]. Due to the possibility of a simultaneous recording of multiple muscles, the EMG activity was one of the first solutions deployed for decoding complex and fine movements of the upper limb, including the hand and fingers [4,5], and great research efforts are still expended toward the full exploitation of EMG signal information to enhance motion and gesture recognition [6,7,8,9,10]. Due to the inherent characteristics of EMG signals that contain information related to motion and are the direct manifestation of the neural commands responsible for the joints’ mechanical actuation, they found a widespread usage also for lower limb prosthetic control [11].

In the latter field, myoelectric data are often used to recognize and predict walking parameters of interest, such as locomotion modes and gait phases, leading to the recognition of a finite number of walking characteristics and movement conditions, constituting valuable information to be included within a wider control framework for driving the behavior of an assistive device and restoring walking functionalities [12,13,14]. However, this kind of approach, essentially based on a classification strategy, is focused on the identification of discrete events that have no temporal development in a continuous fashion, and thus, are not suited for generating smooth and continuous motion patterns [3,12]. On the other hand, myoelectric activity of lower limb muscles is also leveraged for enacting proportional control of a prosthetic device by adopting a regression approach instead of a classification one, with EMG signals that serve as the physiological input reflecting the user’s intended motion, which are then to be decoded into a continuous target parameters [11,15]. The physical quantities estimated from the myoelectric activity of lower limbs typically include joints’ angular trajectories, which are used for producing natural and smooth gait kinematics as the output, with the aim of restoring healthy walking patterns as much as possible [3]. Given the importance of enabling the powered device to shape its response according to the user’s motion intention in an efficient and appropriate way, many works were devoted to enhancing the performance and accuracy of EMG-driven proportional control systems, with a focus on single joints or on the entire lower limb chain, highlighting the key role of myoelectric signals as a human–machine interface for achieving the volitional regulation of prosthetics and assistive devices [16,17,18,19].

Since the ability of a prosthetic device to mimic natural motion dynamics as a response to human commands is one of the main feature the device has to possess [3], it should be kept in mind that during motor tasks that include physical interaction with the environment, movement kinematics is not the only characteristic that is worth considering to ensure a proper description and assessment. This is particularly true for locomotion, where the force exchanged with the ground during the stance phase of the gait possesses a typical and repeatable shape in its three components, and accounts for many aspects related to walking dynamics, such as weight acceptance, progression stability, and propulsion for swing initiation [20]. Currently, the recognized gold standard for ground reaction force (GRF) measurement are dynamometric force plates; this, however, imposes some challenges in terms of costs and the limitation of the measurement environment, where it is not suitable for use outside laboratories. These issues motivated a growing amount of research aimed at proposing approaches for GRF estimation based on alternative technology. Some prior studies proposed methodologies to estimate the GRF from sensors that provide measures of physical quantities closely related to the GRF, such as pressure insoles [21], load cells placed within the shoe [22], or by integrating these kinds of probes with external devices that provide kinematics information [23]. For the same purpose, other works investigated the use of wearable devices since they offer the possibility of continuous and long-term applicability. In this view, inertial measurement units (IMUs) represent an available choice for applications not confined within an instrumented environment, and thus, have been largely employed for GRF estimation during walking [24,25,26].

It is important to note that none of the previous studies included a quantitative evaluation of the possibility to estimate the GRF from EMG signals, although, as mentioned earlier, the latter remain at present the favored human–machine interface for enabling prosthetic regulation [2,11], and in general, very few studies put effort into assessing the reliability of GRF estimation based on myoelectric activity of the lower limb, but not during walking [27]. However, since the value of taking into account dynamics information for powered prosthetic control has been acknowledged [28], the possibility to include myoelectric-based information related to the GRF into the control loop of a powered prosthesis appears attractive since it would be obtained without the need for additional sensors by leveraging the same biosignal used for kinematics estimation. This can potentially lead to a performance improvement of an EMG-driven assistive or supporting device with a more physiological modulation of gait mechanics, in particular during the stance phase, and thus, enhance the naturalness and efficiency, which represent major control objectives in this field [3,28].

In this work, we aimed at assessing the feasibility of estimating the vertical component of the GRF (VGRF) during unconstrained walking by a purely EMG-based approach, i.e., relying only on the myoelectric activity of lower limb muscles. We considered the VGRF since it is the component with the highest magnitude during walking, exceeding the body weight, along with some gait events with a key role in ensuring that stability and propulsion could be clearly recognized in the VGRF, e.g., the weight acceptance during the transition from the swing to the loading response phase, and the need for pushing the body forward at the end of the stance [3,20,29]. To achieve the objectives of this study, we globally considered six lower limb muscles from both the thigh and shank, and we compared the performances of two regression models, i.e., a long short-term memory (LSTM) model and a hybrid model. An extensive evaluation on which muscular compartment and which single muscle would be the most suited for VGRF estimation was also pursued in order to explore the viability of a minimal recording setup.

The rest of this paper is organized as follows: Section 2 provides the methodological framework of this study, reporting information about the data collection and processing (Section 2.1), the regression models and experiments designed for the VGRF estimation (Section 2.2 and Section 2.3), and the outcomes’ quantification (Section 2.4). The main results are reported in Section 3 and discussed in Section 4. The limitations of this study are detailed in Section 5, and the concluding remarks in Section 6 end this paper.

## 2. Materials and Methods

### 2.1. Experimental Setup and Data Processing

**Figure 1:**
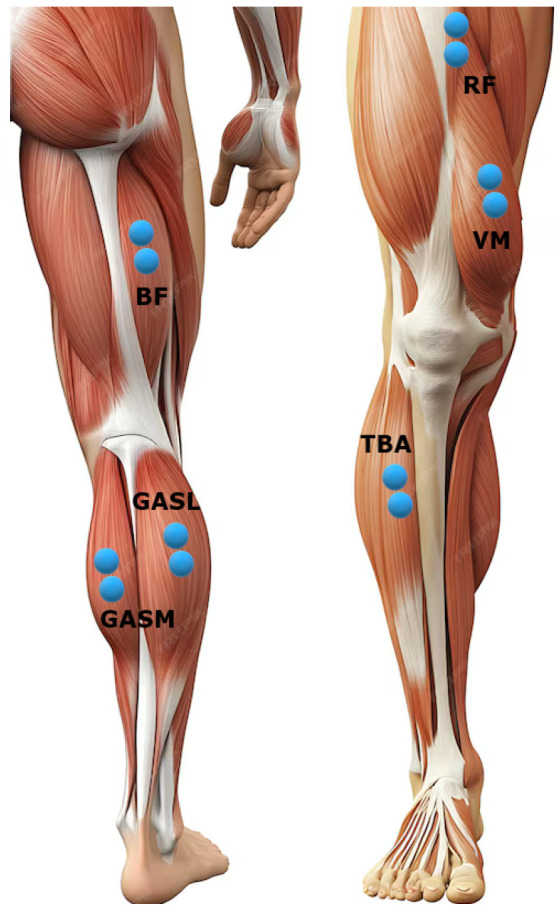
Graphical representation of EMG probes’ placements.

For this study, we enrolled five non-disabled individuals (two males and three females), with an average age of 35.3 ± 10.4 years. The participants were made aware of the purposes of this study and of the experimental procedures, and they provided written informed consent prior to the beginning of the recording session. The dominant lower limb of each volunteer was instrumented with six wireless surface EMG probes (FREEMG system, BTS Bioengineering, Milan, Italy) placed in correspondence with the rectus femoris (RF), vastus medialis (VM), biceps femoris (BF), gastrocnemius medialis (GASM) and lateralis (GASL), and tibialis anterior (TBA). A visual representation of the probes’ placements is provided in Figure 1. The subjects were instructed to walk for about 5 min on an 11 m straight walkway without any external imposition regarding speed or cadence. When the subjects reached one end of the walkway they were requested to turn around and keep walking without any break in order to limit the loss of gait pace.

**Figure 2:**
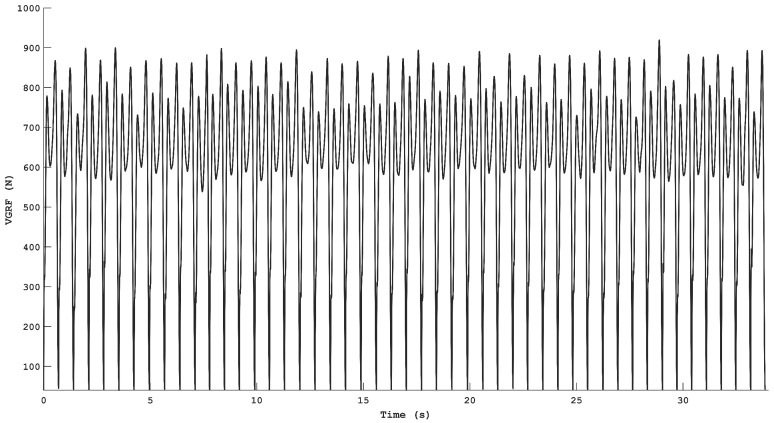
Measured VGRFs during an entire experimental trial for one of the enrolled subjects. Note that for representative purposes, the temporal epochs without force plate recordings were removed and the VGRFs are depicted in a continuous fashion.

Force data were collected by using dynamometric force plates, which represent the gold standard for this kind of data recording. In detail, six force plates (P-6000, BTS Bioengineering, Milan, Italy) were located in the center of the walkway and measured 400 × 600 mm each. The force plates could act as a continuous instrumented floor of 800 × 1800 mm, which allowed the subject to not modify their pace when impacting with the foot on a single plate. Each volunteer performed four gait trials, and for every passage on the force plates, a maximum of two stance intervals were recorded. Thus, for every subject, the VGRF data relative to about 50 stance periods were available for each gait trial (Figure 2). The number of gait trials was chosen as a tradeoff between having enough data for implementing an independent training/testing scheme (see Section 2.3) and avoiding the insurgence of muscular fatigue due to prolonged walking sessions. The experimental activity was performed at the Movement Analysis Laboratory of the Università Politecnica delle Marche (Ancona, Italy).

**Figure 3:**
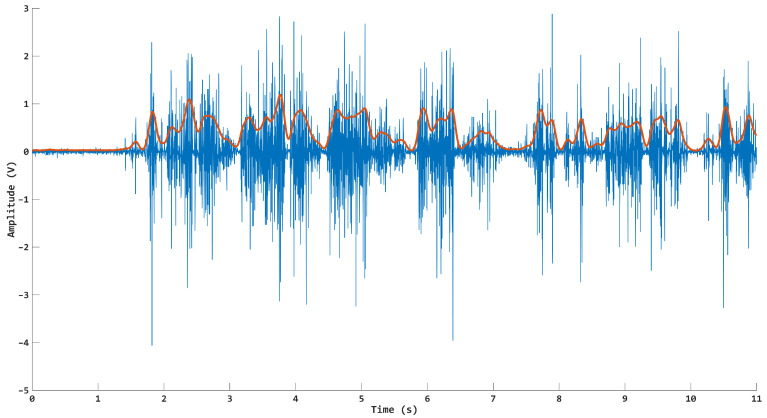
A limited temporal epoch of a raw EMG signal (blue line) with the relative envelope superimposed (red line).

The myoelectric and force raw signals were sampled at 1000 Hz by using the same measurement system, which provided synchronized recordings without the need for any off-line alignment post-processing. The EMG data were band-pass filtered (30–450 Hz) by a 4th-order Butterworth digital filter, whereas the VGRF data were low-pass filtered with a cut-off frequency of 15 Hz. Force data were also normalized with respect to the body weight (BW), which allowed for pooling together the results relative to different subjects. Then, we computed the linear envelope from all six EMG channels for the whole-gait trials by the method proposed in [30], and used this as the input for the regression models (Figure 3). This kind of feature is one of the most favored when dealing with joint kinematics estimation for both upper and lower limbs, with fast extraction and low computational demands [17].

### 2.2. Regression Models

As the regression model for VGRF estimation, we relied on an LSTM network since it represents a deep learning architecture successfully employed for many different EMG-based applications that involved both classification and regression problems [10,16]. LSTM was proposed by Hochreiter and Schmidhuber [31] to address the issue of long-term dependency in sequential data highlighted by recurrent neural networks (RNNs) that can lead to the vanishing of the gradient. The latter is due to the fact that for RNNs, the backpropagation also takes place over time, and the gradient can vanish according to the increase in the time instants, leading to a negligible change in the network weights, negatively impacting the training process. In this way, RNNs are able to handle only short-term memory information.

In general, an LSTM unit has three inputs, i.e., the $x_{t}$ that accounts for the current instant, the cell state $c_{t-1}$ that accounts for the long-term memory, and the hidden state $h_{t-1}$ that accounts for the short-term memory. Both of the latter come from the LSTM of the previous instant. The inner structure of an LSTM is composed of four fully connected layers: the forget gate F, the input gate I, the candidate memory cell C, and the output gate O. These layers update the cell state and hidden state, thus regulating the information flow within the unit. Based on $x_{t}$ and the hidden state content at t−1, the forget gate F establishes the amount of information from the cell state at t−1 that is retained in the current cell state $c_{t}$. On the other hand, the amount of new data to be included in $c_{t}$ is governed by I and C, also in this case on the basis of $x_{t}$ and $h_{t-1}$. The update of the cell state is thus performed as follows:

(1) $$\begin{array}{c} c_{t}=F⊙c_{t-1}+I⊙{\hat{c}}_{t} \end{array}$$

where ⊙ indicates the Hadamard product, and ${\hat{c}}_{t}$ is a candidate cell vector. Then, the now-updated $c_{t}$ is used for computing $h_{t}$ by the output gate O, receiving as input $h_{t-1}$ and $x_{t}$:

(2) $$\begin{array}{c} h_{t}=O⊙\tanh(c_{t}) \end{array}$$

Thus, an LSTM unit updates the long-term memory, i.e., the cell state, by using the short-term past information ($h_{t-1}$) and new information ($x_{t}$). Then, the short-term memory, i.e., the hidden unit, is updated by the long-term memory. Lastly, both $c_{t}$ and $h_{t}$ are passed as input to the LSTM unit at t+1. For the LSTM layers, the state activation function was the hyperbolic tangent, and the gate activation function was a sigmoid. The input weights were initialized by using the methodology proposed in [32], whereas for the recurrent weights, the orthogonal function was used [33].

**Figure 4:**
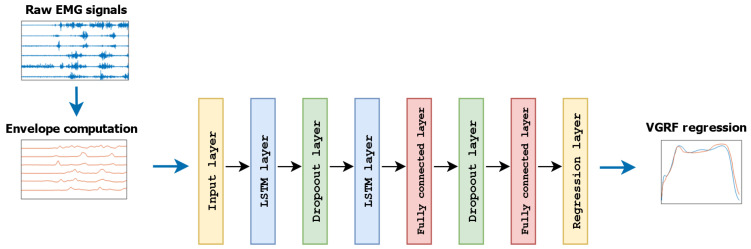
Main structure of the first deep learning network used for VGRF estimation (LSTM_*net*_).

In this work, we exploited two different LSTM-based networks used for estimating the VGRF during walking, starting from the general structure proposed in some previous studies [16,34]. We decided to rely on such kinds of architecture because of their ability to learn long-term dependencies within the data, which is particularly attractive when dealing with the regression of physical quantities that possess cyclical similarities, as happens for muscular activity during a stereotyped and repetitive task, such as walking. This was confirmed by previous works, where LSTM-based networks were used for estimating angular joint kinematics during walking [11,16]. More in detail, the first network (LSTM_*net*_) was developed starting from the structure proposed in previous studies facing EMG-based classification and regression problems [10,16]. The main structure of LSTM_*net*_ included two LSTM layers with 50 hidden units each, two dropout layers with a probability of 0.5 as regularization to prevent overfitting, and two fully connected layers (Figure 4).

**Figure 5:**
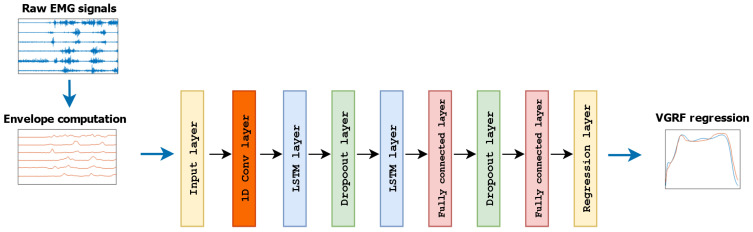
Main structure of the second deep learning network used for VGRF estimation (CLSTM_*net*_).

The second architecture we leveraged was a hybrid deep learning model [19,34] based on a modified version of LSTM_*net*_, where a 1D convolutional layer was included before the long short-term units (CLSTM_*net*_, Figure 5). In brief, a 1D convolutional layer performs the convolution operation by a sliding kernel, which is a set of weights tuned during the training, and the output value was obtained by multiplying each element of the input by the corresponding element of the kernel:

(3) $$\begin{array}{c} \left(x⊗w\right)\left(i\right)=\sum_{j=0}^{k-1}x\left(i+j\right)\cdot w\left(j\right) \end{array}$$

where x(i) denotes the input signal, w(j) is the kernel, and *k* is the kernel size. We included the convolutional layer in the front end of the CLSTM_*net*_ structure in order to highlight local patterns within the data, and thus, leverage the sliding convolutional filters applied to the single dimensional input we considered in this study in order to extract hidden features from the high-level data. These latter features were then used for feeding the LSTM part of the network, with the purpose of providing more meaningful and structured information instead of directly using the EMG envelope, as in the case of LSTM_*net*_. Unlike 2D convolutional layers, which are leveraged mainly for images, 1D convolutional layers have found widespread usage for sequential data with a single spatial dimension, such as a time series. Although many previous works that dealt mainly with joints kinematics estimation exploited 2D convolutional neural networks (CNNs) as regression architectures [15], 1D CNNs present some advantages that make them preferable for 1D sequential data, mainly related to the their lower computational complexity and faster training. Indeed, the computational demands are significantly higher when performing 2D convolution since a k×k kernel is required over n×n dimensions, with a complexity of the order of $k^{2}\cdot n^{2}$, whereas 1D convolution complexity is of the order of k·n [35]. This makes 1D CNN, and in general 1D convolution, more suitable for being embedded within hardware that has to run in real time and for low-cost applications [19,36]. The convolutional layer we employed had 16 filters with a kernel size of 3 and a step size of 1. These values were based on previous studies that dealt with similar problems related to the regression of physical quantities [34,36].

### 2.3. Regression Experiments

In this work, we set up two different experiments in order to evaluate the performances of LSTM_*net*_ and CLSTM_*net*_ when different inputs were provided to the regression models. In the first experiment (Experiment-I), LSTM_*net*_ and CLSTM_*net*_ were fed the EMG activity of all the recorded muscles (TH-SH configuration), and the thigh (TH configuration) and shank muscles (SH configuration) in a separate fashion. The latter was motivated by the fact that the thigh and shank muscles govern the angular displacement of different joints, thus showing characteristic activation patterns during the walking cycle [37,38] in order to cope with specific biomechanical requirements, especially during the stance phase of the gait [37]. Thus, the purpose of the first experiment was to assess the feasibility of obtaining a reliable estimation of the GRF during free walking by relying only on the myoelectric activity of the lower limb, as well as when less information is available for training, while maintaining a physiological and functional meaning in the subtraction of information at the same time. It is noteworthy that, in addition, the first experiment allowed for exploring the possibility to adopt a reduced recording setup for force prediction during walking, which represents a major objective to be pursued in the field of human movement estimation from EMG signals [18].

The importance of the latter aspect in real scenarios motivated the second experiment (Experiment-II), which was designated in order to push toward the development of a minimal EMG probe setup and explore the feasibility of using a single muscle for VGRF estimation. Hence, in the second experiment, we retained the best model between LSTM_*net*_ and CLSTM_*net*_ as the regression architecture, and then it was fed with the myoelectric activity of each single lower limb muscle, which led to the evaluation of six different muscular configurations, i.e., the TBA, GASM, GASL, BF, RF, and VM.

Regarding the outcomes of the experimental evaluations, we proposed two main hypotheses, listed below. Concerning the first experiment, we assumed that the TH-SH configuration would show better outcomes with respect to the TH and SH configurations since a greater amount of input information was provided to the regression models in terms of the EMG signals. This hypothesis appeared reasonable also from a functional viewpoint since considering the thigh and shank muscles together meant including the whole lower limb chain activity within the estimation framework instead of only its proximal or distal parts, thus entailing more detailed information about the lower limb dynamics and its contribution in shaping the VGRF during the stance period. At the same time, it sounds plausible that the TH configuration would offer lower performance with respect to the SH one given that the latter includes muscles that specifically regulate the ankle joint, and thus, have a direct impact on the force exchange with the ground. Based on the abovementioned considerations, the hypothesis we proposed regarding the second experiment was that GASM and GASL would outperform not only the thigh muscles but also the TBA given their specific roles in controlling the dynamics of the stance phase of the gait [37].

For each experiment, we followed the same strategy for training and testing the LSTM_*net*_ and CLSTM_*net*_ regression models. Given that each volunteer performed four gait trials (see Section 2.1), we decided to follow a 50–25–25 scheme for the dataset division, where half of the data were allocated for training (2 gait trials), 25% of the data were reserved for validation (1 gait trial), and another 25% of the data for testing (1 gait trial). Such a scheme was iterated for every subject until each gait trial was used at least once for training, validation, and testing, which allowed for limiting possible issues related to overfitting and data leakage since the models were tested on totally unseen data belonging to a completely different gait trial. For the loss function to be minimized during training and validation, we used the root-mean-square error between the measured and the predicted VGRF. The optimization algorithm was the adaptive learning rate algorithm (Adam), with an initial learning rate of 0.01, a gradient threshold of 1, and a batch size of 200. We set the maximum number of iterations equal to 100 because during the preliminary assessment, we noticed that there was no further significant decrease in the loss with a higher number of iterations. In order to ease the models’ training, the input and target data were scaled by the *z*-score normalization, which provided zero-meaned and unitary variance data.

### 2.4. Evaluation of Regression Performances

The performances of the force estimation were evaluated by means of the following metrics that are commonly used for the quantification of the estimation error. We computed the coefficient of determination ($R^{2}$) as an index for quantifying the goodness of fit of the regression model [17,18]:

(4) $$\begin{array}{c} R^{2}=1-\frac{\sum_{i}{\left(y_{i}-{\hat{y}}_{i}\right)}^{2}}{\sum_{i}{\left(y_{i}-\bar{y}\right)}^{2}} \end{array}$$

where $y_{i}$ and $\bar{y}$ denote the measured VGRF and its average value, respectively, whereas ${\hat{y}}_{i}$ represents the output of the regression model. Furthermore, the root-mean-square error (RMSE) between the estimated and target data was also considered [17,23], defined as follows:

(5) $$\begin{array}{c} RMSE=\sqrt{\frac{1}{n}\sum_{i=1}^{n}{\left(y_{i}-{\hat{y}}_{i}\right)}^{2}} \end{array}$$

where *n* indicates the total number of samples. Note that RMSE values are reported as the percentage of BW (see Section 2.1). We also included the normalized RMSE since it provides the magnitude of the error with respect to the measurement range [25,26,39]:

(6) $$\begin{array}{c} NRMSE=\frac{\sqrt{\frac{1}{n}\sum_{i=1}^{n}{\left(y_{i}-{\hat{y}}_{i}\right)}^{2}}}{max(Y)-min(Y)}\cdot 100 \end{array}$$

where *Y* represents the VGRF measured by the force plate. As an additional metric, we considered the waveform distortion (WD), which quantifies the intensity of distortion between the estimated and the measured VGRF since the GRF waveform has a characteristic and repeatable shape whose estimation error merits to be quantified also in terms of distortion, as happens for other human-motion-related quantities, such as angular kinematics [40]:

(7) $$\begin{array}{c} WD=\sqrt{\frac{1}{n}\sum_{i=1}^{n}{\left(\epsilon_{i}-\bar{\epsilon }\right)}^{2}} \end{array}$$

where $\epsilon_{i}$ denotes the residuals between the estimation and the measurement, and $\bar{\epsilon }$ denotes their average value. Considering the characteristics of this performance metric, the WD values are reported as normalized with respect to the subject body mass (N/kg).

The results are provided as an average over all the subjects with the relative standard deviation. Pairwise statistical comparisons were performed with a paired *t*-test (Mann–Whitney *U*-test) in the case of normally (not normally) distributed data. Multiple comparisons were performed by the ANOVA test (Kruskal–Wallis test) in the case of normally (not normally) distributed data, followed by Tukey’s post hoc test. Normality was assessed by the Kolmogorov–Smirnov test. Statistical significance was set as α=0.05.

## 3. Results

### 3.1. Experiment-I

**Figure 6:**
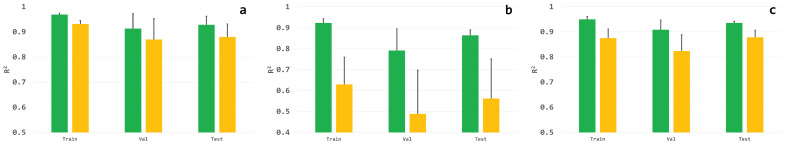
$R^{2}$ values for the CLSTM_*net*_ (green bars) and LSTM_*net*_ (yellow bars) models obtained for the training, validation, and testing phases for the TH-SH (**a**), TH (**b**), and SH (**c**) muscle configurations.

The estimation performances for the TH-SH, TH, and SH muscular configurations in terms of the $R^{2}$ of LSTM_*net*_ and CLSTM_*net*_ are reported in Figure 6 for the training, validation, and testing. No performance drops were observed, with almost constant outcomes that showed in any case better values for CLSTM_*net*_ with respect to LSTM_*net*_, which provided the lowest performances for the TH modality, with values that exceeded 0.6 only in testing. Table 1 reports the regression results for the testing data, confirming that CLSTM_*net*_ offered better performances in providing VGRF estimation from myoelectric signals for all the muscular configurations. This was further supported by the statistical evaluations since the differences between LSTM_*net*_ and CLSTM_*net*_ for all the metrics reported in Table 1 in the TH-SH, TH, and SH configurations were statistically significant (p<0.005 for $R^{2}$, RMSE, NRMSE and WD).

**Figure 7:**
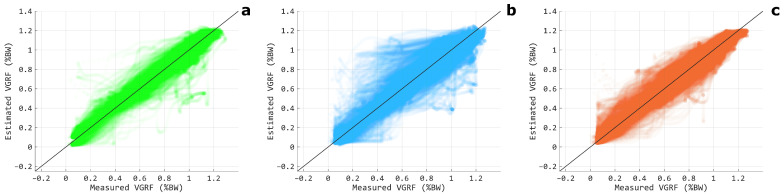
Scatter plots of the measured VGRF versus the correspondent estimation for the TH-SH (**a**), TH (**b**), and SH (**c**) muscular configurations. The black lines denote theoretically perfect regressions. The data are relative to CLSTM_*net*_ and to all the tested subjects.

**Figure 8:**
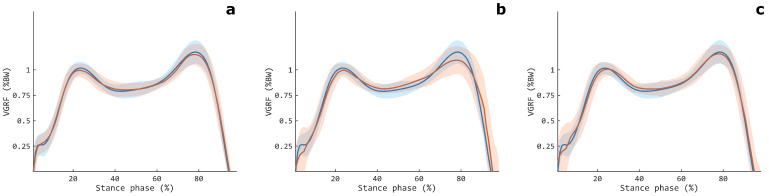
Measured (blue line) and estimated (red line) VGRFs obtained in testing, averaged among all the volunteers, for the TH-SH (**a**), TH (**b**), and SH (**c**) muscular configurations. The shaded areas denote the standard deviations. The data are relative to CLSTM_*net*_.

Regarding the comparison between the three muscular configurations, the TH modality showed the lowest performances, as confirmed by the statistically significant differences observed for all the considered estimation metrics (p<0.001 for both LSTM_*net*_ and CLSTM_*net*_). Conversely, we obtained comparable results for the TH-SH and SH modalities, with a statistically significant difference between the two muscle configurations for both CLSTM_*net*_ and LSTM_*net*_. A scatter plot representation of the VGRF estimation for the TH-SH, TH, and SH configurations is reported in Figure 7, where the TH modality shows a more spread out distribution around the bisect with respect to the TH-SH and SH configurations, whose superior suitability for estimating the GRF appears confirmed. Figure 8 depicts the actual and estimated VGRFs obtained in testing and averaged among all the recorded stance phases and subjects.

Overall, the results of Experiment-I highlight two main points: first, CLSTM_*net*_ outperformed LSTM_*net*_ for all the considered muscular configurations, likely indicating that the introduction of a convolutional layer allowed for extracting high-level hidden features that effectively aided the LSTM blocks’ capability to learn long-term dependencies within the data. For this reason, CLSTM_*net*_ was retained as the regression model for Experiment-II. Second, we observed no performance drop when only the shank muscle was preserved as the regression input, supporting the opportunity to perform an analysis focused on each single muscle, as was achieved in Experiment-II.

### 3.2. Experiment-II

**Figure 9:**
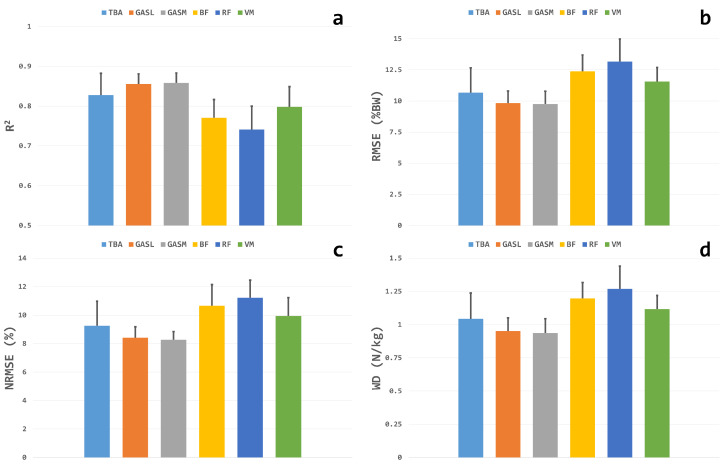
VGRF estimation metrics obtained for the testing data for each single muscle configuration. Panel (**a**) reports the $R^{2}$ values; panel (**b**) contains the RMSE; and panels (**c**,**d**) show the NRMSE and WD values, respectively.

**Figure 10:**
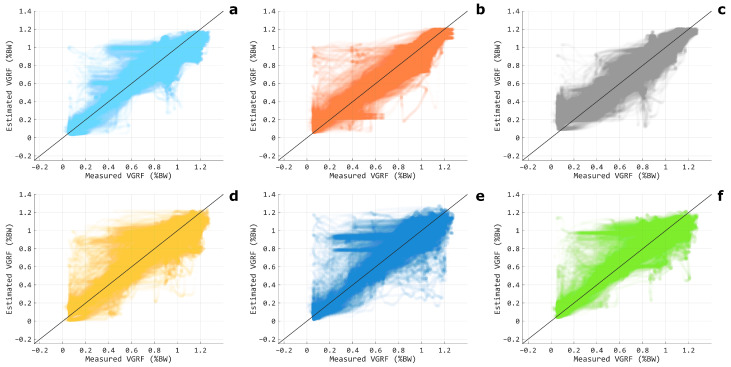
Scatter plots of the measured VGRF versus the correspondent estimation for each single muscle configuration: TBA (**a**), GASL (**b**), GASM (**c**), BF (**d**), RF (**e**), and VM (**f**). The black lines denote theoretically perfect regressions. The data are relative to CLSTM_*net*_ and to all the tested subjects. The color coding is in accordance with Figure 9.

**Figure 11:**
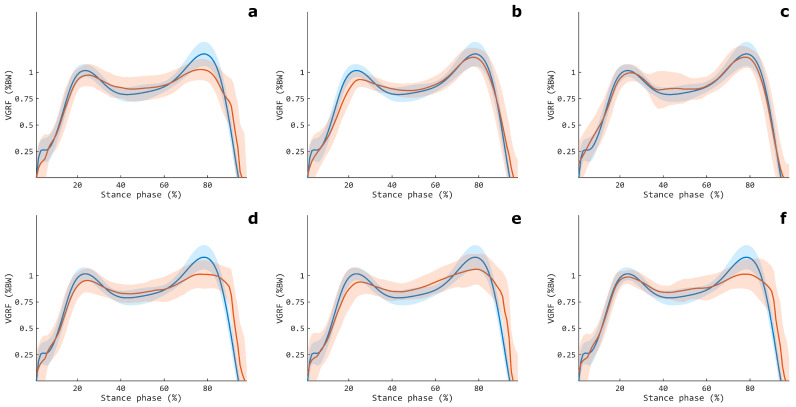
Measured (blue line) and estimated (red line) VGRF obtained in testing, averaged among all the volunteers, for each single muscle configuration: TBA (**a**), GASL (**b**), GASM (**c**), BF (**d**), RF (**e**), and VM (**f**). Shaded areas denote the standard deviations. The data are relative to CLSTM_*net*_.

The estimation outcomes relative to each single muscle configuration are depicted in Figure 9. Overall, the shank muscles provided the best regression outcomes, confirming what was observed in Experiment-I. No statistically significant differences arose between the TIB, GASL, and GASM for any of the considered regression metrics, whereas the lower performances of BF and RF with respect to all the three shank muscles in terms of $R^{2}$, RMSE, NRMSE, and WD resulted in statistical significance (p<0.01). On the other hand, the configuration that involved only the VM muscle did not show any significant difference with respect to the TBA, GASL, or GASM (p>0.05). The VGRF estimation against the measured values is reported in Figure 10 for each single muscle configuration. Figure 11 depicts the actual and estimated VGRFs obtained during testing, averaged among all the recorded stance phases and subjects.

## 4. Discussion

### 4.1. Experiment-I

In this work, we aimed at assessing the feasibility of VGRF estimation during free walking by relying solely on lower limb muscle activity. For this purpose, we compared two deep learning regression models: one based on the LSTM architecture, while the other one was a hybrid model. Overall, the outcomes of the first experiment suggested that these kinds of regression models based on long short-term memory units were effective for the objective of this work, where they produced $R^{2}$ values well above 0.8 (Figure 6) without any performance drop when applied to unseen data in testing, which allowed us to rule out overfitting issues. The suitability of LSTM-based models aligns well with many previous studies, where similar architectures were leveraged for the estimation of kinematics quantities during walking [16,17,41], likely because of their ability to learn temporal dependencies within the data that are a specific feature of periodic human movements, such as walking, where for each gait cycle, the joint kinematics and GRF are expected to show cyclical behavior.

On the other hand, Experiment-I also showed that CLSTM_*net*_ outperformed LSTM_*net*_ in terms of all the considered estimation metrics (Table 1), with significantly better outcomes (Section 3.1). This indicates that the introduction of a convolutional layer in the front end of the main structure (Figure 4 and Figure 5) offered critical advantages in terms of the VRGF estimation accuracy, with decreases in RMSE, NRMSE, and WD well above 20% with respect to LSTM_*net*_ (Table 1). The superior performances of CLSTM_*net*_ were further recognized when the thigh and shank muscles were tested separately, where the beneficial effects given by the CLSTM_*net*_ were particularly evident when observing the outcomes relative to the TH modality. Indeed, CLSTM_*net*_ dramatically raised the estimation performances, where it achieved a level that was at least comparable with the TH-SH and SH modalities, whilst LSTM_*net*_ failed in providing reliable force estimation (Table 1). The SH configuration showed a similar behavior, where CLSTM_*net*_ offered significantly better outcomes for all the considered metrics (Table 1), highlighting once more the greater reliability of this regression model in producing a stable and accurate estimation of the VGRF during walking. It is worth noticing that the estimation enhancement given by CLSTM_*net*_ was not limited to the estimation metrics’ values but was also consistent in terms of the variability, with a reduced standard deviation for all the considered parameters (Table 1), pointing out a more stable estimation among different users with respect to LSTM_*net*_. All these points support the introduction of a block performing convolution before the LSTM units for the purposes of this study, indicating that additional meaningful information can be extracted from the EMG envelope instead of using the latter as the direct input for the LSTM blocks [17].

Regarding Experiment-I, the first hypothesis we made (Section 2.3) was actually rejected. Indeed, the TH-SH configuration did not provide the best performances among the other muscular configurations since the SH modality showed estimation outcomes not significantly different with respect to the TH-SH, and in some cases, even slightly better (Table 1). This indicates that muscles with direct control on the ankle joint can be considered without the need for also including thigh muscles, whose addition did not significantly raise the VGRF estimation goodness. Furthermore, the shank muscles alone should be favored over the thigh ones since, coherently with the previous scenario, the TH configuration offered statistically significantly lower performances with respect to the SH one (Table 1). This can also be appreciated from Figure 7, where the scatter plot of measured and estimated VGRF for TH-SH and SH modalities were closer to the diagonal with respect to the TH configuration, which, in turn, showed more spread samples, denoting greater differences between estimation and measurement, and thus, lower accuracy.

The abovementioned findings appears in agreement with the functional biomechanics of the lower limb, considering that the TH muscles mainly regulate the knee joint kinematics, thus exerting only an indirect impact on the force exchange with the ground. Instead, SH muscles are the primary muscles responsible for the foot movement and ankle kinematics, with the TBA mainly active during the heel strike and early stance, whereas the GASL and GASM are recruited during the mid- and late stance in order to control the single-leg stance and to help with the toe-off [37,38]. In this way, SH muscle activation patterns cover about the entire stance phase of the gait, likely allowing a more accurate mapping of their myoelectric activity to the VGRF. Indeed, the SH configuration showed an estimation close to the actual value for the entire stance phase, also in terms of variability, and in line with that of the TH-SH modality (Figure 8). On the other hand, the TH offered, on average, poorer performances, in particular during the late stance (from about 80% of the stance phase). This could have been due to its predominant role during the initial period of the stance, where it contributes to maintaining knee joint extension for the heel strike and weight acceptance, whereas it shows very limited activity during the rest of the stance [38].

The goodness of the results provided by the TH-SH and the SH modalities was further strengthened by a comparison with existing solutions for GRF estimation from the EMG activity. Indeed, our estimation outcomes outperformed those presented in [27], where an RMSE of 7.33% for the VGRF estimation during unperturbed balance and not lower than 16% for the stepping motion task were produced. Notably, very similar results were obtained when the thigh and shank muscles were used together, and with shank muscles only, agreeing with our observation that including the thigh muscle data as the input did not provide significant advantages for the GRF estimation.

Furthermore, our results appear fully comparable with existing state-of-the-art solutions that mainly employ technological solutions not based on myoelectric activity. Currently, the majority of the works that deal with GRF estimation are based on inertial systems. Multiple lower limb locations for a single inertial sensor were tested in [29], which showed errors not lower than 10% (RMSE) and 7.15% (NRMSE). An approach based on a multiple-IMU setup for joints moments and GRF estimation was proposed in [42]. Although, in one case (three IMUs), they showed an NRMSE slightly lower than our best case (5.49 ± 4.36%), the associated variability was quite high when compared with our results (4.36% versus 0.40% for the SH modality), highlighting that the use of EMG for GRF estimation provides stable results when applied to different subjects. The methodology we propose also maintains validity against a full-body IMU setup, such as that in [24], with an error of 5.3% for normal walking. Although slightly lower than what we obtained for the SH modality (Table 1), this value was achieved by using 17 IMU sensors, which represents an instrumental setup that is hardly feasible for practical applications, while in the present work, similar results were obtained by relying only on three EMG probes.

Our results also remain consistent when compared with previous studies where different signals were used for the GRF estimation. In [21], an RMSE of 6.40% was found for VGRF estimation during straight walking from pressure insoles, in line with the 6.58% we obtained with the SH modality (Table 1). Our methodology also outperformed [22], where three load cells under the foot were employed, reporting an RMSE greater than 65 N for VGRF, whereas the RMSE of CLTSM_*net*_ we obtained corresponded to about 40 N for the TH-SH and SH configurations, and to about 58 N for the TH configuration. An NRMSE of 7.5% for walking at a preferred speed was reported by [23], where six force sensors under the foot were used. Although all the previous works showed good results, it should be highlighted that the estimation was based on sensors that produced signals with a close physical relation with the GRF, which likely allowed for a more direct and easier mapping to the GRF itself. Furthermore, this also highlights the potential of myoelectric signals for being used for a wide spectrum of applications [2], and in particular for estimating physical quantities of different natures that arise during movement that are not limited to joint kinematics [11,18]. A brief summary of previous works is reported in Table 2.

Overall, the outcomes we obtained in Experiment-I indicate the possibility to use the information of few and localized muscles for VGRF estimation during walking. This aspect is central in the field of the volitional control of assistive and rehabilitative devices since using a low number of probes would allow for keeping a lightweight recording setup, enhancing the patient’s comfort and device usability [18,41]. The estimation pipeline we investigated also has potential practical applicability since myoelectric activity still represents the preferred way for developing human–machine interfaces devoted to the volitional control of powered prosthetic and robotics devices [3,11]. This would allow for the integration of a module for the continuous estimation of GRF within a wider framework, enhancing the output of myoelectric-based regulation and producing more physiological walking dynamics.

### 4.2. Experiment-II

The promising results gathered with a limited number of probes in Experiment-I motivated our efforts in investigating the usage of a single EMG electrode for the VGRF estimation in Experiment-II, pushing toward the limit of a minimal setup that would enhance user comfort by lowering the equipment obtrusiveness. In general, each single muscle provided lower performances compared with the TH-SH and SH configurations ($R^{2}$ below 0.9 and NRMSE not under 8%), which was an easily expected result since a significant amount of information was neglected with respect to the previous multi-muscle modalities. This can also be appreciated from Figure 10, where the scatter plots showed a greater spreading around the mid-line when compared with the TH-SH and SH configurations (Figure 7). Still, the TBA, GASM, and GASL muscles outperformed the BF, RF, and VM (Figure 9), where they matched what was observed in Experiment-I regarding the SH modality (Table 1).

On the other hand, our hypothesis that the direct comparison between the triceps surae muscles and TIB would favor the former should be thoroughly reviewed. Indeed, the slightly lower performances of the TIB with respect to the GASM and GASL (Figure 9) were non-significant (Section 3.2), suggesting similar capabilities as input data for force estimation. Nevertheless, unlike the GASL and GAM, the TBA showed an underestimation of the last phase of the stance (Figure 11). This was in line with the functional behavior of the TBA, whose activation pattern was almost entirely focused on the very initial stance and late swing, showing only rare activation bursts in the central part of the gait cycle at most [37]. Thus, the triceps surae muscles might be preferred over the TBA, whose potential for being used alone for VGRF estimation deserves to be further investigated given the abovementioned absence of significantly lower performances in terms of the estimation metrics.

Similar considerations can be expressed for the thigh muscles, whose failure in offering accurate VGRF estimation was evident near the second peak of the VGRF, which corresponded to the push-off. Again, this appears fully coherent with their activation patterns being mainly devoted to knee joint control during the heel strike and late swing [44]. Therefore, the usage of one of the investigated thigh muscles for a minimal recording setup seems to be not recommended at present. However, although it did not reach the performance level of the shank muscles, the VM offered the best results among the thigh muscles, close to those of the TBA (Figure 9) and comparable with those presented in some previous studies. For instance, the markerless system proposed in [39] provided an NRMSE of about 10% while requiring an instrumented environment for data recording. This indicates room for further efforts committed to the enhancement of thigh muscle performances, and the VM in particular, in order to provide a technical solution for VGRF estimation during gait that is also suitable for those subjects whose shank muscles are severely compromised, as in the case of lower limb amputees [3].

The main findings of this work are summarized in the following: The myoelectric activity of lower limb muscles was fully suited for enabling the reliable estimation of VGRF during walking, with the potential to be integrated within an EMG-driven framework for prosthetic volitional control. A regression model that included convolutional and LSTM blocks proved to be a convenient architecture for the purposes of this study, likely because of its hybrid structure that allowed for exploiting the extraction of hidden features from the input, together with the learning of long-term structures within the data. Lastly, the in-depth analysis carried out on the lower limb muscular compartments revealed that the shank muscles alone provided very promising outcomes in terms of the VGRF estimation, without any significant improvement given by the inclusion of the thigh muscles. This would potentially allow for adopting a minimal recording setup, with the possibility to further reduce the number of EMG probes if needed by relying on the triceps surae muscles only.

## 5. Limitations and Future Work

As a final note, it is fair to report that this work had some limitations that merit discussion, also representing grounds for future research. Here, we focused on the vertical component of the GRF only due to its predominant role during the gait in terms of the magnitude, but the methodology we proposed deserves to be tested also on the other force components in order to achieve a complete 3D GRF estimation by relying only on EMG signals. In addition, future studies should investigate the suitability of myoelectric activity for predicting lower limb joint moments that represent important information not only for enhancing the volitional control of assistive and robotic devices but also for providing lightweight and portable systems for gait analysis. In this view, the potential of the methodology we propose should also be explored by considering different types of locomotion modes and terrains, e.g., on inclined surfaces, stairs ascending/descending, turning, and also during running. We are also planning to test the online suitability of the force estimation from EMG signals in order to assess the feasibility of an actual integration within a kinematics estimation system, thus providing a wider solution for kinetics estimation from EMG signals. Lastly, few words deserve to be spent about the limited sample size of the population considered in this study. Although five subjects were enrolled, the total number of analyzed stance periods was above one thousand, which allowed the results to be grounded and reliable, even if with an inter-subject variability that merits improvement. Indeed, it is fair to state that the full generalization of the present findings requires the latter to be verified on a wider population, possibly also including patients with lower limb impairments since their muscular activity and patterns can be different with respect to those of non-disabled individuals. Indeed, this latter point is of paramount importance when dealing with the myoelectric-based control of assistive devices since the target users are amputees and non-able-bodied individuals, whose type and degree of impairment can vary significantly, and each of them would require the careful testing of the proposed solutions in order to ensure proper adaptation. In this work, we focused on healthy subjects to assess the feasibility of estimating the VGRF only from lower limb EMG, but we recognize that the present results require extension and validation on data collected from a wider cohort composed also by non-intact limb patients, possibly with different kinds of impairment.

## 6. Conclusions

In this work, we investigated the suitability of lower limb myoelectric activity for estimating VGRF during walking. The TH-SH and SH configurations showed very similar results, pointing out the possibility to use a reduced recording setup. On the other hand, the use of a single muscle configuration did not reach the same level of performance, where it requires additional efforts in order to exploit a single EMG probe for force prediction. The outcomes were fully comparable, and in some cases, even better than those exhibited by state-of-the-art methods that leverage inertial information from IMU sensors, and also with respect to the use of pressure and force insoles. One of the key points of the solution we proposed is being purely EMG-driven, and thus, with the potential of being integrated within a module for the volitional regulation of powered rehabilitative and prosthetic devices.

## Figures and Tables

**Table 1:** Comparison between CLSTM_*net*_ and LSTM_*net*_ for the three muscular configurations considered in the first experiment. The results are relative to the testing data.

Configuration	Model	R^2^	RMSE (%BW)	NRMSE (%)	WD (N/kg)
TH-SH	LSTM_*net*_	0.88 (0.05)	8.71 (1.90)	7.38 (1.29)	0.84 (0.19)
	CLSTM_*net*_	0.93 (0.03)	6.73 (1.48)	5.76 (1.09)	0.65 (0.14)
TH	LSTM_*net*_	0.56 (0.19)	16.94 (4.16)	14.27 (2.65)	1.63 (0.39)
	CLSTM_*net*_	0.86 (0.03)	9.55 (1.07)	8.20 (0.83)	0.93 (0.10)
SH	LSTM_*net*_	0.88 (0.03)	8.99 (1.10)	7.62 (0.78)	0.86 (0.09)
	CLSTM_*net*_	0.94 (0.01)	6.58 (0.40)	5.65 (0.50)	0.63 (0.04)

**Table 2:** Summary of the VGRF estimation performances of previous works. Straight walking at a preferred speed was considered. When multiple configurations were present, the best result is reported.

Reference	Signals	Estimation Method	Error Metric	Error Value
Sakamoto et al. [27]	10 EMG probes	LSTM	RMSE	7.33%
Karatsidis et al. [24]	17 IMUs	Biomechanical model	NRMSE	5.3%
Jiang et al. [29]	1 IMU	Random forest	NRMSE	7.15%
Hossain et al. [42]	3 IMUs	CNN	NRMSE	5.49%
Liu et al. [43]	1 IMU	Biomechanical model	NMAE	5.81%
Shahabpoor and Pavic [25]	1 IMU	Biomechanical model	NRMSE	5.6%
Tan et al. [26]	4 IMUs	Self supervised learning	rRMSE	8.2%
Eguchi and Takahashi [21]	15 pressure insoles	Gaussian process	NRMSE	6.4%
Kim et al. [22]	3 uniaxial load cells	LSTM	RMSE	65.12 N
Oubre et al. [23]	6 force sensitive resistors, knee angle sensor	Random forest	NRMSE	7.5%
Sugai et al. [39]	Motion capture	LSTM	NRMSE	9.88%

LSTM: long short-term memory; RMSE: root-mean-square error; NRMSE: normalized RMSE; NMAE: normalized mean absolute error; rRMSE: relative RMSE.

## Data Availability

Data can be made available upon reasonable request.

## Abbreviations

The following abbreviations are used in this manuscript:

BF	Biceps femoris
BW	Body weight
CNN	Convolutional neural networks
EMG	Electromyography
GASL	Gastrocnemius lateralis
GASM	Gastrocnemius medialis
GRF	Ground reaction force
IMU	Inertial measurement unit
LSTM	Long short-term memory
NRMSE	Normalized root-mean-square error
RF	Rectus femoris
RMSE	Root-mean-square error
TBA	Tibialis anterior
VGRF	Vertical ground reaction force
VM	Vastus medialis
WD	Waveform distortion
